# Locating previously unknown patterns in data-mining results: a dual data- and knowledge-mining method

**DOI:** 10.1186/1472-6947-6-13

**Published:** 2006-03-07

**Authors:** Mir S Siadaty, William A Knaus

**Affiliations:** 1 Department of Public Health Sciences, University of Virginia School of Medicine, Box 800717, Charlottesville, Virginia, 22908, USA

## Abstract

**Background:**

Data mining can be utilized to automate analysis of substantial amounts of data produced in many organizations. However, data mining produces large numbers of rules and patterns, many of which are not useful. Existing methods for pruning uninteresting patterns have only begun to automate the knowledge acquisition step (which is required for subjective measures of interestingness), hence leaving a serious bottleneck. In this paper we propose a method for automatically acquiring knowledge to shorten the pattern list by locating the novel and interesting ones.

**Methods:**

The dual-mining method is based on automatically comparing the strength of patterns mined from a database with the strength of equivalent patterns mined from a relevant knowledgebase. When these two estimates of pattern strength do not match, a high "surprise score" is assigned to the pattern, identifying the pattern as potentially interesting. The surprise score captures the degree of novelty or interestingness of the mined pattern. In addition, we show how to compute p values for each surprise score, thus filtering out noise and attaching statistical significance.

**Results:**

We have implemented the dual-mining method using scripts written in Perl and R. We applied the method to a large patient database and a biomedical literature citation knowledgebase. The system estimated association scores for 50,000 patterns, composed of disease entities and lab results, by querying the database and the knowledgebase. It then computed the surprise scores by comparing the pairs of association scores. Finally, the system estimated statistical significance of the scores.

**Conclusion:**

The dual-mining method eliminates more than 90% of patterns with strong associations, thus identifying them as uninteresting. We found that the pruning of patterns using the surprise score matched the biomedical evidence in the 100 cases that were examined by hand.

The method automates the acquisition of knowledge, thus reducing dependence on the knowledge elicited from human expert, which is usually a rate-limiting step.

## Background

In the twentieth century, computers began making it possible to store huge amounts of data through mass digital storage. It soon became clear that for stored data to be used, database management systems were needed. Database management systems (DBMS) help a user to locate and retrieve specific data/information in a timely manner [1]. During the past decade, the size of databases, however, has exceeded the capacity of scientists, engineers, and other experts to analyze them. This means most data are currently being stored in a "write-only" mode, an acknowledgement of their unlikely use and analysis [2]. Automating the data analysis process with data mining is one possible approach [3]. The majority of data mining methodologies, however, produce large amounts of patterns from the data [4,5]. Among these mined patterns there are many that are previously known, or not interesting. Ideally one would like to prune the uninteresting patterns, and present a refined subset to the human expert.

Numerous methods have been proposed to filter the results of data mining, and identify the subsets of those rules that would be more interesting for the user. Hilderman and Hamilton provide the Heuristic Measures of Interestingness, a set of such methods from the literature, all of which are based on diversity [6]. Silberschatz and Tuzhilin classify measures of interestingness into two classes, the objective and the subjective measures [7]. Objective measures rely on the internal structure of the data and mined patterns (measures such as confidence, support, gain, chi-squared value, gini, entropy gain, laplace, lift, and conviction [8]), while subjective measures try to capture some domain-relevant knowledge from a human expert or user, and then use it either to prune the uninteresting patterns [9] or to identify interesting ones. Liu et al. have proposed methods for post-processing of mined rules, where the user provides the knowledge with three degrees of preciseness using a simple specification language: general impressions, reasonably precise concepts, and precise knowledge [10].

Objective measures act mainly as a filter so that subjective measures can be more efficiently applied [11]. Subjective measures can create bottlenecks because they rely on human experts to provide subjective knowledge, but such human experts may be unavailable. Knowledge elicited from a human expert may need to be coded in a specialized and task-specific format/language, and its updating, expansion, and reuse may be difficult [12]. To address the disadvantages of relying on the user to elicit knowledge and the human expert to provide knowledge for the subjective measures of rule interestingness, we propose to design and implement an automated process that would systematically identify interesting patterns from the large pool of mined patterns, by cross-examining results of database mining with a relevant knowledgebase. With this approach, there is a reduced need to acquire knowledge from the user. This method for the discovery of novel and interesting patterns is based on comparing the strength of patterns mined from a clinical database with the strength of the equivalent patterns mined from a relevant knowledgebase. When these two estimates of strength do not match, a high "surprise score" is assigned to the pattern, identifying the pattern as potentially interesting. This process addresses the limitations of human experts when performing data mining on biomedical databases.

In this paper we present the dual mining method. Also, we report preliminary results from implementing a pilot version.

## Methods

### A. Overview

**Figure 1:**
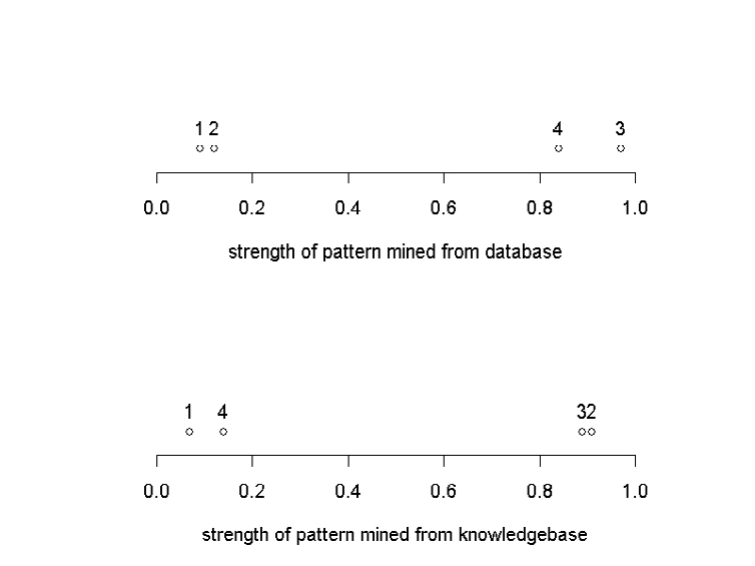
DB and KB strengths of four patterns.

**Figure 2:**
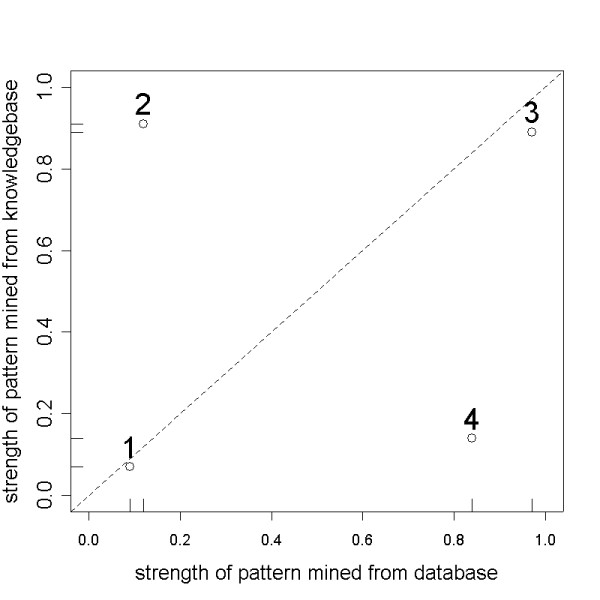
Simultaneous strengths of four patterns.

Consider four patterns mined from a database (DB), where their strengths (of association), on a scale of 0 to 1, are 0.09, 0.12, 0.97, and 0.84. The top graph of Figure 1 shows the strengths of these four patterns, where each pattern is shown by a point. Patterns 3 and 4 are obviously the stronger ones. As previously indicated, however, a strong pattern might already be known and therefore not very interesting or potentially useful. To determine which of the patterns could be interesting we next estimate the strengths of similar patterns in a knowledgebase (KB) containing human knowledge pertinent to that database. The bottom graph of Figure 1 illustrates the strengths of the four patterns in the knowledgebase, where patterns 2 and 3 are the strong ones. Comparing the results of these two associations derived from DB and KB mining, one can see that pattern 1 is weak in both DB and KB, pattern 2 is weak in DB but strong in KB, pattern 3 is strong in both DB and KB, and pattern 4 is strong in DB but weak in KB. If we treat the DB and KB strengths of each pattern as its coordinates in a two-dimensional space, one can produce a scatterplot where the DB and KB numbers are shown simultaneously, as in Figure 2.

In Figure 2, the diagonal 45-degree line is where the DB and KB strengths are the same. Patterns close to the diagonal are not of much interest to the user, but patterns that are farther away from the diagonal line may be interesting. Therefore it is distance from the diagonal, rather than the strength of a pattern, that defines its degree of interestingness. For example, pattern 4 is a strong pattern mined from the DB, but there is not that much current knowledge supporting it, so it may point to a potential discovery. Pattern 2 appears to be well-established knowledge, but the association estimated in the database is not large, a surprise finding that may be worthwhile investigating further.

### B. The algorithm

We begin with two digital repositories, one a database (DB) and the other a knowledgebase (KB) relevant to that database. Table 1 is the algorithm for the dual-mining method. In biomedicine, databases usually use "aggregate classifications" as their terminology, which have a balanced number of exhaustive, mutually exclusive, stable categories. And knowledgebases usually use "detailed clinical vocabularies" that provide specific codes for each concept, incorporate discoveries quickly, and have multiple hierarchies. One needs to be able to map the attributes to both DB and KB terminologies.

**Table 1:** The dual mining algorithm

1	Given a database (DB) to be mined, select a relevant knowledgebase (KB);
2	Produce a mapping of terminology of DB to KB, and/or vice versa. In biomedicine, databases usually use "aggregate classification", while knowledgebases usually use "detailed clinical vocabularies";
3	Choose "primary unit of analysis" for DB and KB. Examples of unit of analysis for DB are each 'patient', each 'visit', or an 'episode of care'; and for KB is each biomedical article;
4	Choose a type of pattern, a mining method for finding that type of pattern, and its measure of pattern strength. For example, association rules where strength of the rule is measured by Spearman's Rho;
5	Given list of attributes, and their sampling probabilities, generate m n-tuples. m and n are integer numbers. m is count of n-tuples that are chosen simultaneously for a single iteration. For example, m can be a number like 20, 50, or 100. n is count of attributes within a pattern. For example, n may range from 2 to 5;
6	Evaluate the batch of m n-tuples in the DB, and estimate strength of each n-tuple;
7	Estimate strengths of the same n-tuples in the KB;
8	Estimate the surprise score (SS), by using the pair of strengths of each n-tuples in DB and KB. Besides, estimate statistical significance of the scores;
9	Update list of sampling probabilities of attributes by using the estimated SS's. Attributes observed more frequently in n-tuples with high SS, will receive higher sampling probabilities, while attributes of low SS n-tuples receive lower probabilities.
10	Start over from step 5, until all n-tuples generated from the list of attributes are exhausted, or the time limit is reached.

For biomedical DBs, such as patient databases, primary units of analysis (the records) may be each visit, each patient (and hence all visits of that patient), or an episode of care consisting of a cluster of visits of the same patient that are all about the same complaint. In biomedical KBs, each document or article may be treated as the primary unit of analysis.

Given the list of attributes (where an attribute is presence/absence of a biomedical sign or symptom), and the type of pattern and mining method, we generate m patterns, where m is an integer, the number of patterns that can be evaluated at one iteration, given the computational power available. If m is large enough such that contains all the possible patterns generated from the attribute list, one iteration will be sufficient. Otherwise, one may use multiple iterations and a simple random sampling scheme, without replacement, to choose m from the list of all possible patterns. Each of the patterns is unique, but they may share some attributes.

Then we estimate the strength of each pattern. For example, if the pattern is a pair of attributes and one wants to measure dependence of the attributes, then one produces a contingency table. For example Table 2 shows a 2-by-2 contingency table for a pattern with two attributes, where each attribute has two possible values (this will be relaxed later). One queries the database to populate the table. For example, one retrieves number of records where Attribute 1 is present and Attribute 2 is present, and the retrieved count would be n1 in the Table 2. Table 3 shows a set of three queries that can populate the 2-by-2 table, given the total number of records of the database (N) is known. Given the three counts Q1 to Q3 returned by queries 1 to 3 of Table 3, the cell counts of the contingency table are n1 = Q3, n2 = Q2 - Q3, n3 = Q1 - Q3, and n4 = N - (Q1 + Q2 - Q3).

**Table 2:** 2-by-2 contingency table

		**Attribute 1**	
		
		present	absent
**Attribute 2**	present	**n1**	**n2**
	absent	**n3**	**n4**

**Table 3:** Set of three queries populating the 2-by-2 table

Query 1	find number of records where Attribute 1 contains value "present"
Query 2	find number of records where Attribute 2 contains value "present"
Query 3	find number of records where Attribute 1 contains value "present" AND Attribute 2 contains value "present"

Then, to measure the strength of dependence of the two attributes, one chooses an association statistic for categorical variables such as Spearman's rho [13]. However, we choose not to use Spearman's Rho. In the case where the cell count n4 in Table 2 and the total table sample size N are very close, Rho may not be an optimal association measure. Rho tends to grow rather slowly when the expected and the observed cell counts for n1 are substantially different under the null hypothesis of independence. This is the usual case when dealing with biomedical knowledgebases such as PubMed. We propose to use the following measure of association:

r_i _= c * LOR_i _/(1 + c * abs(LOR_i_))     (1)

where r_i _will be a number between -1 and 1, with 0 meaning no association, -1 meaning strong inverse association, and 1 strong direct association. In formula 1, LOR_i _is the log-odds-ratio of the table for pattern i, defined as log(n1*n4/n2*n3). abs(.) is the absolute value function. c is a scale factor, typically smaller than 1. For example, choosing c to be 1/2, r_i _reaches 0.5 (half its maximum strength) when the LOR is 2, or equivalently when the OR is exp(2) = 7.4; hence c determines the speed with which r_i _grows. Hence, one can correct the weakness observed in Rho.

Next, we query the knowledgebase to populate the same contingency table, and measure the strength of association for the same pattern. Then we feed the two association estimates of each pattern, one from DB and the other from KB, into a function that computes a single number, the surprise score (SS). In formula (2)

SS_i _= (r_DBi _- r_KBi_)^k^/2^k ^    (2)

r_DBi _and r_KBi _are the association estimates for pattern i mined from the database and the knowledgebase respectively. When k is two, formula (2) gives a quadratic surprise function, where SS grows nonlinearly. An alternative is choosing an odd number for the power k, so that the sign of the relationship of DB and KB associations is preserved. 1/2^k ^is a normalizing constant, a scale parameter, such that the range of SS is standardized. Given k = 2, the constant would be 1/4, so that the range of SS would be from 0 to 1.

In computing the SS, an implicit assumption is that, for majority of patterns, association measures r_DBi _and r_KBi _are the same, within some random noise. This in turn means that the distribution of the r_DBi _association measures should be fairly similar to the distribution of the r_KBi _association measures. However, in a particular application of the dual-mining method, the two distributions could be systematically different, such as a shift in location or a difference in scale. Before computing the SS, one normalizes the two scores so that they have the same center and dispersion. For example, under a general location-scale family

r_NormedDBi _= a + b * r_DBi _    (3)

where r_DBi _is the original association measure estimated for pattern i from DB, and r_NormedDBi _is the normalized association. a is the location and b is the scale corrections. Then one computes SS_i _using r_NormedDBi_, as shown in formula (2). Factors a and b are estimated by comparing the distribution of r_DBi _with r_KBi_. Using a robust measure of central tendency, such as Huber's m-estimator, one estimates the center of the two distributions [14]. Then one subtracts the two estimated centers to get an estimate of a. Also, one estimates a robust measure of dispersion, such as the inter-quartile range (IQR) of each of the two distributions, and then divides them to get an estimate of b.

To attach p-values to each SS, testing if it is significantly different from zero, one estimates a non-parametric distribution for them using a bootstrap approach, such as the bias-corrected and accelerated non-parametric method [15]. Then one builds confidence intervals, and computes estimates of variation enabling hypothesis testing.

Using magnitude of the computed SS, plus its significance of p-value, one sorts the patterns, and chooses the ones with highest surprise, denoting high interestingness. To combine the magnitude of SS with its statistical significance for the purpose of ranking, one method is to generate a 95% confidence interval (CI) for each SS, then select the CI bound that is closer to zero, and use it in the ranking. For n-tuples (a pattern or rule that is composed of n attributes) where CI contains zero, one uses zero as its SS value. One then generates a report on the selected patterns for inspection by human agent.

Given the attribute list, if all the possible patterns were not evaluated in one iteration, or if one prefers to evaluate patterns in smaller batches of size m, one needs to start over with a new batch of n-tuples. Note each new iteration of the dual mining algorithm covers disjoint groups of n-tuples. Furthermore the results of previous iterations, the surprise scores of previously evaluated n-tuples, are not changed during later iteration. Using multiple passes of the algorithm may have advantages. Given the intractability of evaluating all possible n-tuples, one may want to generate n-tuples for a new iteration that seem to have better chance of being interesting. To do so, one may use results of previous iterations, to characterize n-tuples with higher surprise scores. For example one can compute the attributes appearing most frequently in the high surprise n-tuples, as well as those appearing most frequently in low surprise n-tuples. These frequencies may be used in the new iteration to generate n-tuples using mostly attributes in the previously high surprise n-tuples, and avoiding attributes in the previously low surprise n-tuples. In other words, one uses the frequencies to update sampling probability of step 5, thus deviating from an equal probability sampling.

### C. Alternatives

To attach p-value to the SS, an alternative approach is to estimate variance of r_DBi _and r_KBi_, and then use formula (2) to compute variance of SS_i _for a single pattern i. Then one builds confidence intervals (CI) and tests the hypothesis that that single SS_i _is significantly different from zero (under a normality assumption). This way, some patterns could be filtered out before the step of bootstrapping described above. A multiple comparison issue may need to be addressed here. To compute variance of r_i _one can start from the known formula for variance of LOR, and then apply the delta method [13]. In the formula

var(r_i_) = var(LOR_i_) * c^2 ^/(1 + c * abs(LOR_i_))^4 ^    (4)

var(r_i_) is variance of the association measure introduced in formula (1), and c is the scale factor of formula (1). var(LOR_i_) is computed based on the four cell counts of the respective contingency table, that is var(LOR) = 1/n1 + 1/n2 + 1/n3 + 1/n4. In the case where surprise score is computed by the formula SS_i _= d * (r_NormedDBi _- r_KBi_), variance of the SS_i _would be

var(SS_i_) = (b^2 ^* var(r_DBi_) + var(r_KBi_))/2^2k ^    (5)

under the independence assumption of r_DBi _and r_KBi_. var(r_DBi_) and var(r_KBi_) are the variances of association strengths for pattern i estimated by formula (4), k is the power of formula (2), and b is the scale factor from formula (3).

The association statistics are usually defined for 2 attributes. To generalize this, measuring strength of dependence for patterns with more attributes, and attributes with more categories, a method is to use log-linear models. For patterns with more attributes than two, one adds more dimensions to the contingency table. For attributes with more categories than two, one adds more rows/columns to the table. One can show that a loglinear model fitted to the cell counts of the n-dimensional contingency table estimates strength of dependence of the n attributes simultaneously [16]. For the 2-by-2 contingency table, the model

log(C_ij_) = S + X_i _+ Y_j _+ XY_ij _    (6)

fits the cell counts C_ij _of row i and column j, by the term S representing the sample size effect, term X_i _representing the probability of row i, Y_j _representing the probability for column j, and term XY_ij _which represents the association between X and Y. Function log(.) is the natural logarithm. Now one extends model (6) to a three-way contingency table, representing a pattern with three attributes (an n-tuple where n = 3). In the model

log(C_ijk_) = S + X_i _+ Y_j _+ Z_k _+ XY_ij _+ XZ_ik _+ YZ_jk _+ XYZ_ijk _    (7)

the term XYZ_ijk _measures the three-attribute association. Likewise, extension of model (7) to n-way contingency table is straightforward, where the table represents an n-tuple, with n being any integer. The range of term XYZ_ijk _is all real numbers, from minus infinity to plus infinity. One may use the transformation used for LOR in formula (1) to normalize the association range from -1 to +1.

One may use other methods of data-mining, besides the association, with the dual-mining process. One only needs to replace the association scores with other measures of strength of patterns, based on that particular data-mining approach.

## Results

We applied the dual-mining method to the University of Virginia's Clinical Data Repository (UVA CDR), as the database [17], and to the National Library of Medicine's PubMed, as the knowledgebase [18]. To access patient data, approval from University of Virginia Human Investigation Committee was obtained (HIC approval # 11946). For this study, we chose two tables of the CDR, the 'diagnoses' and the 'labs'. During each patient visit, one or more diagnoses are assigned to the patient. These diagnoses are coded according to the International Classification of Diseases (ICD) standard, and saved in the CDR diagnoses table. For each patient visit, zero or more diagnostic lab tests are ordered by the doctor. Results of these lab tests are saved in the CDR labs table. There are several thousand lab types and diagnoses in the CDR. We used frequency of attributes as one of the selection criteria when forming the attribute list, so that the statistical process would have maximum power to separate signal from noise.

CDR uses aggregate classifications such as ICD-9 to code and save data, while PubMed articles utilize detailed clinical vocabularies. We mapped the CDR terminology to the PubMed, using Medical Subject Headings (MeSH) and free-text medical terminology [19]. Accuracy of the mapping was a second factor to select the attributes. This resulted in 96 disease attributes, and 105 lab attributes. Table 4 shows a partial attribute list, ten disease and ten lab attributes. Note some lab attributes are 'derived'. For example 'anemia' of row 12 is defined as values of lab 'hematorcrit' that are below a certain cutoff.

**Table 4:** Attribute list sample

	**Ont_S_**	**Ont_KB_**	**Ont_DB_**
1	hypertensive disease	hypertension	disease::40$|40$.%%
2	malignant hypertension	malignant hypertension	disease::401.0
3	benign hypertension	benign hypertension	disease::401.1
4	hypertensive heart disease	hypertensive heart disease	disease::402|402.%%
5	secondary hypertension	secondary hypertension	disease::405.%%
6	type 2 diabetes mellitus	type 2 diabetes mellitus OR diabetes mellitus type 2	disease::250.00
7	type 1 diabetes mellitus	type 1 diabetes mellitus OR diabetes mellitus type 1	disease::250.01
8	diabetic ketoacidosis	diabetic ketoacidosis	disease::250.1%::4
9	heart failure	heart failure	disease::428|428.%%
10	supraventricular tachycardia	supraventricular tachycardia	disease::427.0
11	hematocrit	hematocrit	lab::HCT|HCTCH|HCTN|HCTSP|HCTSH|HCTPM::999::cdr
12	anemia	anemia	lab::HCT|HCTCH|HCTN|HCTSP|HCTSH|HCTPM::3::cdr
13	polycythemia	polycythemia	lab::HCT|HCTCH|HCTN|HCTSP|HCTSH|HCTPM::7::lab
14	blood glucose	blood glucose OR blood sugar	lab::GLUC|GLCPOC|GLUCI|CMGLUC|GLUCI#2|GLUCN|GLUCF::999::cdr
15	hypoglycemia	hypoglycemia	lab::GLUC|GLCPOC|GLUCI|CMGLUC|GLUCI#2|GLUCN|GLUCF::3::cdr
16	hyperglycemia	hyperglycemia	lab::GLUC|GLCPOC|GLUCI|CMGLUC|GLUCI#2|GLUCN|GLUCF::7::lab
17	blood urea nitrogen	Blood urea nitrogen OR bun	lab::BUN|PreBUN|PostBUN|BUN#2::999::cdr
18	low blood urea nitrogen	(Blood urea nitrogen OR bun) AND (low OR decreas*)	lab::BUN|PreBUN|PostBUN|BUN#2::3::cdr
19	high blood urea nitrogen	(Blood urea nitrogen OR bun) AND (high OR increas*)	lab::BUN|PreBUN|PostBUN|BUN#2::7::cdr
20	blood potassium	potassium/blood	lab::K|KIC|KN|Q1288::999::cdr

We constructed all possible pairs composed of one disease attribute and one lab attribute. This generated 10080 n-tuples (where n = 2). Besides the first batch of 10080 n-tuples, we ran the algorithm four more times (iterations), each time evaluating the n-tuples in a different sex-race subset of the data. We chose two sexes (male, female) and two races (black, white), and composed all the possible combinations. Thus we constructed an additional 10080 * 4 = 40320 n-tuples where n = 3. Hence, collectively we evaluated 50400 n-tuples where n = 2 or 3. Since we evaluated all possible n-tuples exhaustively, we used 'equal probability' sampling for all the iterations.

For the mining methodology, we used 'associations'. We measured strength of association by the statistic given in formula (1). To estimate association between the disease and the lab in each n-tuple, we used all years of CDR data, from 1993 to 2005. We used each patient visit as the primary unit of analysis for CDR, and used each article as the primary unit of analysis for PubMed. We estimated strength of association of each n-tuple using 27.5 million lines of CDR data (containing data from 9.4 million visits), and 15.7 million articles of PubMed.

When normalizing the r_DBi _and r_KBi _scores, we weighted them by the inverse of their variances. Hence, data points with more uncertainty would affect the normalization process less. The estimated normalization shift factor was 0.6513 - 0.4918 = 0.1595 (using Huber's m-estimator), that was subtracted from the r_KBi _scores. The estimated normalization scale factor was (0.5375 - 0.4412)/(0.6826 - 0.5988) = 1.1492 (using IQR), that was multiplied into the r_KBi_.

When ranking the n-tuples according to their surprise score, we used the method of constructing 95% CI and choosing the CI-bound closer to zero, hence incorporating both magnitude and significance of the SS in the ranking process.

To query CDR and PubMed we wrote routines in Perl [20]. Also, for data preprocessing, and for passing results between different routines (as a 'glue') we used Perl. We implemented the statistical routines in R [21]. Both Perl and R are open source free software.

**Figure 3:**
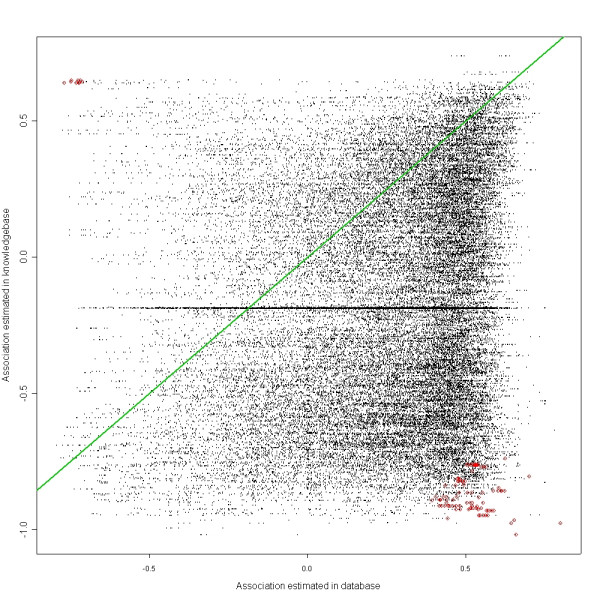
Pairs of associations mined from database and knowledgebase.

Figure 3 shows the correspondence between the two association scores of each n-tuple, the score estimated in the CDR versus the score estimated in the PubMed. The diagonal line of uninterestingness is off the center mainly due to zooming on the portion of the graph where points are located. Nutples with top 100 SS's are shown with red circles. Note some points are not in the top 100 even though they appear to be farther away from the diagonal line compared to some red circles. This is due to incorporating statistical significance besides the magnitude of distance from the diagonal. In other words, such n-tuples have larger variance, hence making their SS less significant. Also, since a weighted normalization procedure has been used, the points seem to be distributed non-homogeneously even after normalization.

**Figure 4:**
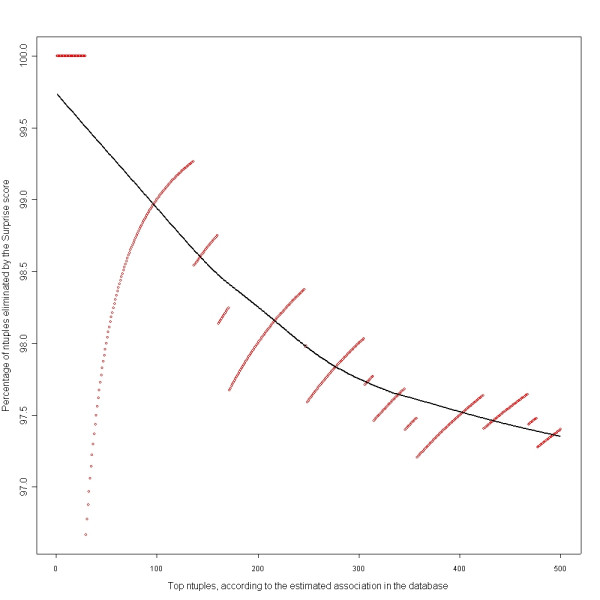
Elimination of uninteresting patterns using the surprise score.

To evaluate how utilizing the surprise score could help prune uninteresting n-tuples, we built list of top n-tuples ranked according to strength of their DB scores, the DB-list. Then we compared the DB-list with the list of ntulpes ranked by the surprise score, the SS-list. Figure 4 shows percentage of n-tuples in DB-lists of different lengths that are not present in the SS-lists of same length (hence pruned). For example, 99% of n-tuples in the DB-list of top 100 are eliminated by using the SS. In Figure 4, the red dots are the observed points, and the solid black curve is a smoother to summarize the trend.

To verify biomedical validity of eliminating uninteresting n-tuples using the surprise score, we inspected top 100 n-tuples of the DB-list, the n-tuples with biggest DB associations. Table 5 shows top twenty n-tuples (column 'DB rank'). In the top 100 list, there were 38 n-tuples where the disease attribute (column 'Attribute 1') is "Routine infant or child health check", where all of them except two have big negative DB scores. Since these are routine checkup visits, one expects that results of labs ordered by the doctor be within normal range. In other words either no lab is ordered (due to benign nature of the visit) or the ordered lab would have normal values. This is in accordance of observing big negative DB associations (column 'r_DBi_'), since a negative association means presence of the disease attribute is associated with absence of pathologic values of labs. Since these are known/obvious facts, the n-tuples won't be interesting to the user, despite their high DB ranks. The estimated associations for the same n-tuples in the KB are negative too (column 'r_KBi_'). Since r_DBi _and r_KBi _are close to each other, the algorithm computes a low surprise score for them, considering them as uninteresting. The ranks according to SS (column 'SS rank') for these n-tuples are in the range of several thousands, showing their general uninterestingness. This is concordant with the biomedical evidence.

**Table 5:** Mined patterns with biggest DB association strengths

	**SexRace**	**Attrbute 1**	**Attribute 2**	**DB rank**	**SS rank**	**r_DBi_**	**r_KBi_**
1	mw	Routine infant or child health check	high blood creatinine	1	36814	-0.793	-0.692
2	tot	Routine infant or child health check	high blood creatinine	2	37629	-0.759	-0.692
3	tot	Routine infant or child health check	hyperprothrombinemia	3	39259	-0.741	-0.738
4	mw	Routine infant or child health check	high blood urea nitrogen	4	36797	-0.760	-0.625
5	mw	Routine infant or child health check	hyperglycemia	5	36059	-0.743	-0.712
6	tot	Routine infant or child health check	high blood urea nitrogen	6	37361	-0.739	-0.625
7	fw	Cardiac arrest	hypernatremia	7	16208	0.722	0.319
8	mb	Routine infant or child health check	high blood creatinine	8	37855	-0.755	-0.692
9	mb	Routine infant or child health check	hyperprothrombinemia	9	38635	-0.780	-0.738
10	fw	acute renal failure	hyperphosph(or|at)emia	10	18058	0.715	0.418
11	fb	Renal osteodystrophy	high blood creatinine	11	22970	0.735	0.528
12	fw	acute renal failure	hypernatremia	12	22502	0.712	0.514
13	tot	Renal osteodystrophy	high blood creatinine	13	23383	0.719	0.528
14	fb	Secondary hyperparathyroidism	blood phosphorus	14	28442	0.717	0.634
15	tot	Cardiac arrest	hypernatremia	15	16649	0.710	0.319
16	tot	Routine infant or child health check	prothrombin	16	36517	-0.718	-0.691
17	mb	Routine infant or child health check	high blood urea nitrogen	17	37201	-0.748	-0.625
18	tot	Secondary hyperparathyroidism	high blood urea nitrogen	18	247	-0.774	0.465
19	mw	Routine infant or child health check	hyperprothrombinemia	19	39155	-0.729	-0.738
20	fw	Routine infant or child health check	high blood creatinine	20	37581	-0.773	-0.692
21	tot	Secondary hyperparathyroidism	hypophosph(or|at)emia	30	7	-0.770	0.640

Other n-tuples of the top 100 DB scores point to strong relationship between a body fluid chemical and a disease, such as hyperphosphatemia, high blood creatinine, or hypernatremia in renal diseases. These are well-known biomedical facts. Therefore treating them as uninteresting is justified biomedically. There is one n-tuple (row 21 of the table) in the top 100 DB-list that attains a high rank of 7 in the SS list (explained below).

We inspected top 100 SS n-tuples to evaluate their biomedical interestingness. There are n-tuples where the lab attribute is not a pathologically low or high value, but whether the doctor ordered the lab or not. We systematically filtered those, as we were more interested in patterns of abnormal body chemicals in different diseases, rather than patterns of medical practice. This eliminated 72 of the top 100 n-tuples. The remaining 28 were repetitions (in different patient subgroups) of the 10 shown in Table 6. This is the reason for gaps in the column 'SS rank'.

**Table 6:** Mined patterns with biggest SS

	**SexRace**	**Attrbute 1**	**Attribute 2**	**SS rank**	**r_DBi_**	**r_KBi_**
1	fw	nephritis	hypercapnia	5	0.571	-0.950
2	tot	Secondary hyperparathyroidism	hypophosph(or|at)emia	7	-0.770	0.640
3	fw	ventricular fibrillation	low serum albumin	33	0.615	-0.859
4	fw	ventricular fibrillation	anemia	45	0.564	-0.772
5	fb	apnea	high serum albumin	83	0.605	-0.848
6	fb	ventricular fibrillation	thrombocytopenia	88	0.626	-0.740
7	fw	ventricular tachycardia	anemia	91	0.521	-0.785
8	fw	sleep apnea	thrombocytopenia	92	0.443	-0.960
9	fw	glomerulonephritis	hypercapnia	93	0.553	-0.867
10	fw	ventricular tachycardia	high serum albumin	99	0.543	-0.884

Top SS n-tuples are of two classes, the ones that have negative r_DBi _but positive r_KBi_, and the reverse. In Table 6 all the n-tuples have positive r_DBi _and negative r_KBi _except one. For row 2, although PubMed articles indicate that hypophosphatemia is a common finding in patients with secondary hyperparathyroidism, in CDR patients this does not happen. We speculate this absence of the expected relation could be due to therapeutic interventions. Treatments tend to return the abnormal lab values toward their normal range.

For the rest of n-tuples (9 out of 100), some relations are observed in CDR patients that have not been published in PubMed articles. We did not find any medical interpretation to rule them out. For example, there is no article explaining elevated blood concentration of CO2 in patients with nephritis. This is what the system found in CDR patients. We believe these are interesting leads worthy of further investigations.

## Discussion

### A. Comparison of methods for measuring pattern interestingness

Considering data-mining as a prime method for analysis of explosively expanding biomedical data and knowledge, it is quite natural to intend to automate the process as much as possible. While numerous researchers have automated the post-processing of the mined rules (using some measures of interestingness), few have automated the knowledge acquisition step. Our proposed method tries to accomplish this. This is a major difference with some of the existing measures of subjective interestingness where the algorithm requires interaction from the user, in one or more of the stages of preprocessing, rule induction, or post-processing [22]. Basu et al describe a method where the knowledge required to evaluate novelty of text-mined rules is acquired automatically [23]. However, their measure of interestingness, the distance between components of the rule in the semantic network of WordNet, is estimated solely in the knowledgebase. In our method, the surprise score utilizes both DB and KB to estimate interestingness. The method of estimating "surprise" by comparing two or more data and knowledge spaces has commonality with methods of "scientific discovery" in cognitive sciences [24].

When mining databases in the field of biomedicine for scientific research purposes, rule "interestingness" has less to do with "user's interests" and more to "novelty" of a mined rule, and whether the rule has been reported previously anywhere in the biomedical literature. Then relying on one or a few user or human expert to supply the domain knowledge, required for selection of interesting rules, is inadequate. There are numerous subspecialties in biomedicine, with continuous addition of new knowledge, facts, and relations. Moreover, in the scientific research, thoroughness of review of previous knowledge is an "axiom", where it is insufficient to rely on user's interests and concepts. Our proposed method not only reduces dependence on human and automates the process, it also makes it possible to incorporate much larger, more accurate, and up-to-date body of knowledge into the rule interestingness measure.

The dual mining method eliminates the need to develop a specific representation language for biomedicine, to elicit user's knowledge. Medical terminology is a large field, and using domain knowledge expressed in this terminology seems more appropriate. Currently there are quite a few biomedical classifications and terminologies used to represent knowledge and data. We believe this approach will facilitate communication using these standardized terminologies, rather than inventing a new one.

Silberschatz and Tuzhilin suggested incorporating interestingness into the data-mining engine that generates the rules, in order to produce interesting rules in the first place. Viewing the user as the source of knowledge, this requires the user to supply all the knowledge in advance, which is a difficult task to do. Instead, our method can easily incorporate interestingness into the process of rule mining, as it is not relying on user's knowledge. One can use the method in an iterative way, where n-tuples are generated in batches, and then evaluated. This enables the system to guide generation of next batch of n-tuples by the surprise scores of previous batches, hence generating rules with higher probability of being interesting.

A strength of the dual mining approach is that, currently, it is hard for some objective and most subjective measures to capture patterns that have very weak or even non-existent associations [25,26]. Usually weak patterns resulting from data mining are discarded, but very weak patterns may be of interest and potential importance. For example, consider the well established positive association between cigarette smoking and lung cancer that is evident through numerous well-designed biomedical studies. If, however, a patient database containing many smokers and lung cancer cases returned no association between the two, this would be worthy of further investigation. The dual-mining strategy has more power to detect such potentially useful weak associations. Note the difference between a weak association, and a negative one. "Weak" signifies magnitude of association while "negative" signifies its direction. A negative association can be potentially strong. Although traditional association rule induction is not suitable to detect strong negative associations, there have been developments to generalize these "market basket" rules to cover both presence and absence of attributes [27]. However, these do not address the weak association instance described above.

As the quantity and quality of databases increase, implementation of the dual-mining method suggested here may greatly decrease the time required to prune results of data mining. Previous work from our team has demonstrated that, while informative, inspecting the results of data mining on large clinical data repositories for potentially novel hypotheses can be prohibitively labor-intensive [28].

### B. Completeness versus interestingness

The relative efficiency of data mining algorithms is an important consideration when comparing various approaches. Ideally, a data mining application would generate all the patterns existing in the database (the "completeness" criterion), as fast as possible. However, linking collections of databases generated by different entities in different geographical locations reproduce supper massive collections of data that could challenge this approach. More importantly, it has more recently been realized that full coverage of all possible patterns in a database in not the ultimate goal, but finding the patterns that are interesting and useful to the user [29,30].

### C. Primary units of analysis

One needs to decide on the primary unit of analysis for each digital repository. For example, for the CDR, each patient may be considered the primary unit of analysis (a record or row). In this case, for example, presence of smoking and diagnosis of lung cancer in the same patient is evidence of association between the two. However, this will not differentiate between an ex-smoker who has 15-year-gap between stopping the smoking and onset of cancer, versus concurrent cancer and smoking case. Using each visit for the primary analysis may improve but would not completely address this challenge as the two attributes of interest might be present in a patient but recorded on separate visits. A clustering of patient visits, based on inter-arrival times, may provide a possible solution. Also, types of diagnoses coded for each patient visit might be incorporated into an algorithm to cluster the visits into more homogeneous "episodes of care".

Likewise, for the PubMed, each PubMed article may be considered as primary analysis unit. In this case, co-occurrence of the two attributes (smoking and heart disease) in the same paper is evidence of association. However, the two concepts could have been used in the paper in separate paragraphs/sections with no relationship claimed between them, hence being less specific. An analysis at the sentence level may provide a more specific method for estimation of the strength of relationship between the concepts, but it is less sensitive, and also requires much more computational power. Methods in the field of 'natural language processing' may provide optimal solutions.

We note when using PubMed as KB, a negative association may mean absence of relation rather than presence of evidence for an inverse relationship. Also, since we are using "co-occurrence" in PubMed to compute the association, it may become hard to discern direction of association by using the co-occurrence method (even at the sentence or title level). Methods of natural language processing may help in this regard. A quicker solution is to use absolute value of r_DBi _association scores, and to shrink all negative values of r_KBi _to zero, and then computing the SS.

### D. Future work

Before presenting any specific pattern identified by the dual mining method to a human expert, one needs to improve on the level of "intelligence" at which various components of the system are implemented. Criteria for the usefulness of a "new" discovery should be more stringent in clinical settings [31].

One should note that using a human expert to validate the dual mining method usually means the expert is using sources of biomedical knowledge extra to PubMed. Then 'false positive' n-tuples, where the system finds them interesting but the expert considers them known facts and hence uninteresting, could have been correctly classified by the system, had the system have access to those extra sources of knowledge such as medical text books. This may be plausible to do in near future.

In the scatterplot of DB and KB associations, the patterns were treated as independent but some of them share attributes. This may point to a "meta-surprise" function where two patterns have very different SS, despite their sharing a majority of attributes.

Ideally one wants to find and document the sentences in PubMed where a specific relationship between concepts A and B is claimed. This might require determining the type of verb used in the sentence to explain the claimed relationship between the attributes. This would require using natural language processing (NLP). Routines written in Java for NLP as part of the UMLS project may be potential starting points thereby providing a mixture of data and text mining technologies [32].

Since the proposed method for automatic selection of interesting patterns utilizes several digital repositories, potentially heterogeneous and distributed data sources, there will have to be standard and formal reconciliation of terminologies of the repositories.

The majority of digital repositories are currently in a relational databases format. It is optimal to be able to utilize the repositories in their relational form, rather than transforming the records to a 'flat file format' [33]. On the other hand, the majority of statistical functions utilize a default flat format for their data input. Transfer of data over the Internet, its speed and its security, needs to be considered too.

Most clinical databases are also usually special-purpose, restricted in access or private, and frequently not interconnected with other databases or the Web. As a result patient data are rarely freely exchanged and must be protected by security and confidentiality technologies and protocols [34].

To assign reasons/potential mechanisms to the surprising patterns, one may utilize the knowledgebase, using open and closed discovery algorithms [35]. For patterns where DB association is nonzero but KB association is almost 0, one can use methods and tools developed in the field of 'literature-based discovery' (LBD). LBD by definition searches for relationships not contained within the existing knowledgebase [36].

Each measure of association (like Pearson's or Spearman's) tries to capture dependence of two or more attributes in a certain way. The degree to which a particular association statistic can measure such dependence is influenced by the nature of that dependence. For example Pearson's r is not suitable to measure quadratic relationship between two attributes. One may want to use several measures where they can capture different trends of dependence. One should note that ultimately such measures capture association, not causation. In other words, claims of causation (or rules with a structure like "IF a THEN b" implying consequence) cannot be based solely on association measures.

Another possible approach to the surprise function for the dual-mining method is through a Bayesian method, where the prior probabilities are constructed by mining the knowledgebase, and the posterior probabilities are estimated by updating the prior measures through mining the database, or vice versa. One could also compare the strength of a pattern during a specific time interval versus other intervals, where more data is added to the database during time. This would be similar to a trend analysis.

## Competing interests

A patent application has been filed for the dual mining method, by authors of this paper.

## Authors' contributions

MSS conceived of the method, carried out its implementation, and drafted the manuscript. WAK participated in design of the method, and drafted the manuscript. Both authors read and approved the final manuscript.

## Pre-publication history

The pre-publication history for this paper can be accessed here:


